# Metabolomic Profiling, In Vitro Antimalarial Investigation and In Silico Modeling of the Marine Actinobacterium Strain *Rhodococcus* sp. UR111 Associated with the Soft Coral *Nephthea* sp.

**DOI:** 10.3390/antibiotics11111631

**Published:** 2022-11-15

**Authors:** Noha M. Gamaleldin, Hebatallah S. Bahr, Yaser A. Mostafa, Bryant F. McAllister, Amr El Zawily, Che J. Ngwa, Gabriele Pradel, Hossam M. Hassan, Usama Ramadan Abdelmohsen, Dalal Hussien M. Alkhalifah, Wael N. Hozzein

**Affiliations:** 1 Department of Microbiology, Faculty of Pharmacy, The British University in Egypt (BUE), Cairo 11837, Egypt; 2 Department of Pharmacognosy, Faculty of Pharmacy, Nahda University, Beni-Suef 62764, Egypt; 3 Pharmaceutical Organic Chemistry Department, Faculty of Pharmacy, Assiut University, Assiut 71526, Egypt; 4 Department of Biology, University of Iowa, Iowa City, IA 52242-1324, USA; 5 Department of Plant and Microbiology, Faculty of Science, Damanhour University, Damanhour 22511, Egypt; 6 Division of Cellular and Applied Infection Biology, Institute of Zoology, RWTH Aachen University, 52074 Aachen, Germany; 7 Department of Pharmacognosy, Faculty of Pharmacy, Beni-Suef University, Beni-Suef 62511, Egypt; 8 Department of Pharmacognosy, Faculty of Pharmacy, Minia University, Minia 61519, Egypt; 9 Department of Pharmacognosy, Faculty of Pharmacy, Deraya University, Minia 61111, Egypt; 10 Department of Biology, College of Science, Princess Nourah Bint Abdulrahman University, Riyadh 11671, Saudi Arabia; 11 Botany and Microbiology Department, Faculty of Science, Beni-Suef University, Beni-Suef 62511, Egypt

**Keywords:** actinomycetes, *Rhodococcus*, PCA, PLS-DA, antimalarial, metabolomics

## Abstract

Malaria is a persistent illness with a great public health concern. To combat this fatal disease, developing effective antimalarial medications has become a necessity. In the present study, we described the actinomycetes associated with the Red Sea soft coral *Nephthea* sp. and isolated a strain that was sub-cultured in three different media (M1, ISP2, and OLIGO). Actinomycete isolate’s phylogenetic analysis of the 16S rRNA gene revealed that it belongs to the genus *Rhodococcus*. In vitro screening of the antimalarial activity for three extracts against *Plasmodium falciparum* was carried out. Non-targeted metabolomics for the chemical characterization of the isolated actinomycete species UA111 derived extracts were employed using high-resolution liquid chromatography–mass spectrometry (LC-HR-MS) for dereplication purposes. Additionally, statistical analysis of the vast LC-MS data was performed using MetaboAnalyst 5.0. Finally, an in silico analysis was conducted to investigate the potential chemical compounds that could be the source of the antimalarial potential. The results revealed that ISP2 media extract is the most effective against *Plasmodium falciparum*, according to antimalarial screening (IC_50_ 8.5 µg/mL), in contrast, OLIGO media extract was inactive. LC-HRMS-based metabolomics identified a range of metabolites, mainly alkaloids, from the genus *Rhodococcus*. On the other hand, multivariate analysis showed chemical diversity between the analyzed samples, with ISP2 extract being optimal. The docking analysis was able to anticipate the various patterns of interaction of the annotated compounds with three malarial protein targets (*P. falciparum* kinase, *P. falciparum* cytochrome bc1 complex, and *P. falciparum* lysyl-tRNA synthetase). Among all of the test compounds, perlolyrine (11) and 3097-B2 (12) displayed the best docking profiles. In conclusion, this work demonstrated the value of the established method for the metabolic profiling of marine actinomycetes using the data from liquid chromatography–mass spectrometry (LC-MS), which helps to streamline the difficult isolation stages required for their chemical characterization. In addition, the antimalarial efficacy of this strain has intriguing implications for future pharmaceutical development.

## 1. Introduction

Natural products have always been regarded as a rich source of prospective drug candidates for the creation of novel naturally based treatments [1]. Macro- and microorganisms that can resist pressure, salt, and temperature extremes make up the taxonomically and biologically varied marine ecosystems. Marine inhabitants have the ability to create distinctive chemicals with innovative medicinal uses that are not present in their terrestrial equivalents [2]. Marine actinomycetes are a diverse collection of aerobic Gram-positive microbes that have been identified as a source of numerous commercially marketed medications as well as novel therapeutic prospects. These bacterial species are prolific makers of new substances with a variety of biological and pharmacological effects, including, among others, anticancer, antioxidant, antibacterial, and enzyme inhibition [2,3]. *Rhodococcus* is a member of the taxonomic group actinobacteria and is a member of the Nocardiaceae family and the order actinomycetales; they are classified as aerobic, Gram-positive, non-motile, non-sporulating, mycolate-containing, and non-cardioform actinomycetes [4,5], which have remarkable metabolic capacity to manufacture common potent antibacterial substances such rhodopeptins, aurachins, lariatins, rhodostreptomycins, and rhodozepinone. Additionally, it has been demonstrated that they can biosynthesize a few indole derivatives with effective inhibitory properties against bacterial biofilms, as well as peptides, antioxidant carotenoids, siderophores, and certain enzymes [1,3]. Malaria is a public health issue that has persisted for a long time and presumably originated in Africa. Globally, it affects 30 million people and claims roughly 2.5 million lives annually, the majority of whom are young children. All endemic regions of the world now have widespread chloroquine-resistant *P. falciparum* strains, and there is evidence of the evolution of resistance against artemisinin-based combination therapies, which are recently used as first-line treatments for *P. falciparum* [6]. Consequently, there is an increasing need to find new medications [7,8]. As a potent and versatile approach that has the advantage of simultaneously analyzing and characterizing multiple active components in complex mixtures without requiring extensive chromatographic work, metabolomics is attracting increasing attention from experts in a variety of fields related to natural products [9]. In light of this, metabolomic profiling based on liquid chromatography–mass spectrometry (LC-MS) techniques has become a common and successful method for examining the chemical complexity of natural sources with minimal experimental effort, enabling fast drug discovery [1]. As part of our ongoing study on actinomycetes generated from marine microorganisms, in this study, the actinomycete associated with the Red Sea soft coral *Nephthea* sp. was isolated, identified, and cultivated in different culture media using the OSMAC technique. To evaluate the efficacy of fermented extracts from *Rhodococcus* species against *Plasmodium falciparum*, antimalarial screening was carried out. To limit the amount of data obtained and find correlation and differentiation among the tested materials, a chemical investigation supported by LC-HRESIMS was conducted, followed by multivariate data statistical analysis (MVDA). This work investigates the biological potential and chemical metabolites of the marine soft-coral-associated bacterium *Rhodococcus* sp. UA111 as a promising source of new therapeutic agents.

## 2. Material and Methods

### 2.1. Soft Coral Collection

The marine soft coral *Nephthea* sp. (1.5 kg) was obtained in March 2021 at a depth of 7 m off the coast of Hurghada on the Red Sea (2,715,048″ N, 334,903″ E). The Invertebrates Department, National Institute of Oceanography and Fisheries, Red Sea Branch, Hurghada, Egypt, was provided with a voucher sample (NIOF730/2021). The soft coral was identified by El-Sayd Abed El-Aziz (Department of Invertebrates Lab., National Institute of Oceanography and Fisheries, Red Sea Branch, 84511 Hurghada, Egypt). The soft coral biomass was transferred to a plastic bag containing seawater and transported to the laboratory. The soft coral specimens were rinsed in sterile seawater, cut into pieces of ca. 1 cm^3^, and then thoroughly homogenized in a sterile mortar with 10 volumes of sterile seawater. The supernatant was diluted in a ten-fold series (10^−1^, 10^−2^, and 10^−3^) and subsequently plated on agar plates.

### 2.2. Strain Isolation

Three different media [M1, ISP medium 2, and Oligotrophic medium (OLIGO) were used for the isolation of the actinobacteria. All of the media were supplemented with 0.2 µm pore size filtered cycloheximide (100 µg/mL), nystatin (25 µg/mL), and nalidixic acid (25 µg/mL). Cycloheximide and nystatin inhibit fungal growth, while nalidixic acid inhibits many fast-growing Gram-negative bacteria [10]. All of the media contained Difco Bacto agar (18 g/L) and were prepared in 1 L of artificial seawater (NaCl 234.7 g, MgCl_2_·6 H_2_O 106.4 g, Na_2_SO_4_ 39.2 g, CaCl_2_ 11.0 g, NaHCO_3_ 1.92 g, KCl 6.64 g, KBr 0.96 g, H_3_BO_3_ 0.26 g, SrCl_2_ 0,24 g, NaF 0.03 g and ddH_2_O to 10.0 L). The inoculated plates were incubated at 30 °C for 6–8 weeks. Distinct colony morphotypes were picked and re-streaked until visually free of contaminants. Thirty isolates were picked upon morphological differences. The isolates were maintained on plates for short-term storage, and long-term strain collections were set up in a medium supplemented with 30% glycerol at −80 °C.

### 2.3. Molecular Identification and Phylogenetic Analysis

The 16S rRNA gene amplification, cloning, and sequencing were performed according to Hentschel et al., 2015, using the universal primers 27F and 1492R. Chimeric sequences were identified by using the Pintail program. The genus-level affiliation of the sequence was validated using the Ribosomal Database Project Classifier. The genus-level identification of all the sequences was conducted with RDP Classifier (-g 16srrna, -f allrank) and validated with the SILVA Incremental Aligner (SINA) (search and classify option). An alignment was calculated again using the SINA web aligner (variability profile: bacteria). The gap-only positions were removed with trimAL (-noallgaps). For phylogenetic tree construction, the best fitting model was estimated initially with Model Generator. RAxML (-f a m GTRGAMMA –x 12345 –p 12345 -# 1000) and the estimated model was used with 1000 bootstrap resamples to generate the maximum-likelihood tree. The sequences of 16S rRNA from the type strains of Rhodococcus species were identified using the NCBI BLAST server implementing the megablast search program with the RhodUA111 16S rRNA sequence as a query. Preliminary phylogenetic analyses were used to identify the Rhodococcus species most closely to RhodUA111. The sequences were obtained from the NCBI database. The sequence alignment and phylogenetic analysis were performed with MEGA11. A multispecies sequence alignment was obtained with Muscle, and the phylogenetic relationships were revealed using Maximum Likelihood with the TN93+G+I substitution model.

### 2.4. Extract Preparation

The strain (low sequence similarity) was cultivated using 3 different production media (M1, ISP2, and OLIGO) liquid approaches to produce 3 organic extracts. The liquid cultures were grown for 10 days at 30 °C while shaking at 150 rpm. The culture was then filtered, and the supernatant was extracted with ethyl acetate while the cells and mycelia were extracted by shaking with methanol for 4 h. The ethyl acetate extracts were stored at 4 °C.

### 2.5. Antimalarial Assay

To determine the antiplasmodial effect of the fungal extracts on *P. falciparum* erythrocytic replication in vitro, the Malstat assay was used as described. To synchronize the NF54 culture, parasites with many ring stages were centrifuged, and the pellet was resuspended in five times pellet volume of 5% *w/v* sorbitol /ddH20 and incubated for 10 min at room temperature. The cells were washed once with RPMI to remove sorbitol and further cultivated. Synchronized ring-stage parasites with 1% parasitaemia of *P. falciparum* NF54 strains were plated in triplicate in 96-well plates (200 µL/well) in the presence of a serial dilution of the extracts dissolved in 0.5% *v/v* dimethyl sulfoxide (DMSO). The parasites were incubated with the extracts for 72 h at 37 °C in the presence of nitrogen-containing 5% O_2_ and 5% CO_2_. The incubation of parasites with DMSO at a concentration of 0.5% alone was used as negative control, and 20% was used as positive control. Afterwards, 20 µL was removed and added to 100 µL of the Malstat reagent (1% Triton X-100, 10 mg of l-lactate, 3.3 mg Tris and 0.33 mg of APAD (3-Acetylpyridine adenine dinucleotide) dissolved in 1 mL of distilled water, pH 9.0) in a new 96-well microtiter plate. The plasmodial lactate dehydrogenase activity was then assessed by adding a 20 μL mixture of NBT (Nitro Blue Tetrazolium)/Diaphorase (1:1; 1 mg/mL stock each) to the Malstat reaction. The optical densities were measured at 630 nm, and the IC_50_ values were calculated from variable-slope sigmoidal dose–response curves using the GraphPad Prism program version 5.

### 2.6. LC-MS Profiling

Three ethyl acetate extracts from the samples were prepared at 1 mg/mL for mass spectrometry analysis. The recovered ethyl acetate extract was subjected to metabolic analysis using LC-HR-ESI-MS, according to Abdelmohsen et al., 2014. An Acquity Ultra Performance Liquid Chromatography system connected to a Synapt G2 HDMS quadrupole time-of-flight hybrid mass spectrometer (Waters, Milford, CT, USA) was used. The positive and negative ESI ionization modes were utilized to carry out the high-resolution mass spectrometry coupled with a spray voltage at 4.5 kV; the capillary temperature was 320 °C, and the mass ranged from *m/z* 150 to 1500. The MS dataset was processed, and the data were extracted using MZmine 2.20 based on the established parameters. Mass ion peaks were detected and accompanied by a chromatogram builder and a chromatogram deconvolution. The local minimum search algorithm was addressed, and isotopes were also distinguished via the isotopic peaks of the grouper. The missing peaks were displayed using the gap-filling peak finder. An adduct search, along with a complex search, was carried out. The processed data set was next subjected to molecular formula prediction and peak identification. The positive and negative ionization mode data sets from the respective extract were dereplicated against the DNP (Dictionary of Natural Products) databases. ChemBioDraw Ultra 14.0 software was used to create the chemical structure drawings.

### 2.7. In silico Molecular Docking Simulations

Molecular Operating Environment (MOE^®^ 2014.0901) software was used to evaluate and examine the possible interactions of the dereplicated molecules (3, 4, 5, 6, 11, and 12) within the crystal structure of 3 target proteins: *P. falciparum* kinase (PfPK5; PDB ID: 1V0P), *P. falciparum* cytochrome bc1 complex (Cyt bc1; PDB ID: 4PD4), and finally, *P. falciparum* lysyl-tRNA synthetase (PfKRS1; PDB ID: 6AGT). The docking results in the form of a docking score (S; kcal/mol), docking accuracy (root-mean-square deviation; RMSD in Å), and binding interactions with various amino acid residues lining the active site were listed (as shown in Table 1 and Table 2). The preparation of the structural formulas of the test molecules and the structure of the target proteins were performed as reported by [11]. The validation of the prepared protein structures was conducted via the re-docking of the co-crystallized ligands with their protein crystal structure obtained from the RSCB protein data bank (https://www.rcsb.org/ accessed on 8 August 2022), and their docking score and RMSD values were within an acceptable range for running docking simulations within the target proteins (as listed in Table 1). The docking simulations were performed according to a docking protocol reported elsewhere [11], and the results are reported in Table 1 and Table 2. The visual inspections of the produced docking poses (10 poses/molecule) for the binding interactions with various amino acid residues lining the active site of 3 target proteins crystal structures are listed in Table 2 and are represented as 2D and 3D diagrams.

### 2.8. ADME Parameters and Drug-Likeness Computational Analysis

SwissADME free web tool for prediction of physicochemical parameters, pharmacokinetics, and drug-likeness of small molecules (http://www.swissadme.ch/ accessed on 7 August 2022), was used to measure such parameters of compounds VIa-Vik, and the results are listed in Table 3. Additionally, the medicinal chemistry filters (Lipinski, Ghose, Veber, Egan, and Muegge) were applied to all of the test compounds, and none violated such filters. PAINS (Pan-Assay of Interference Compounds) were used to examine the specificity of such a class of compounds as possible anticancer agents, and all compounds yielded zero scores, as shown in Table 3.

## 3. Results and Discussion

### 3.1. Identification of Red Sea Soft Coral Associated Actinobacteria 

**Figure 1:**
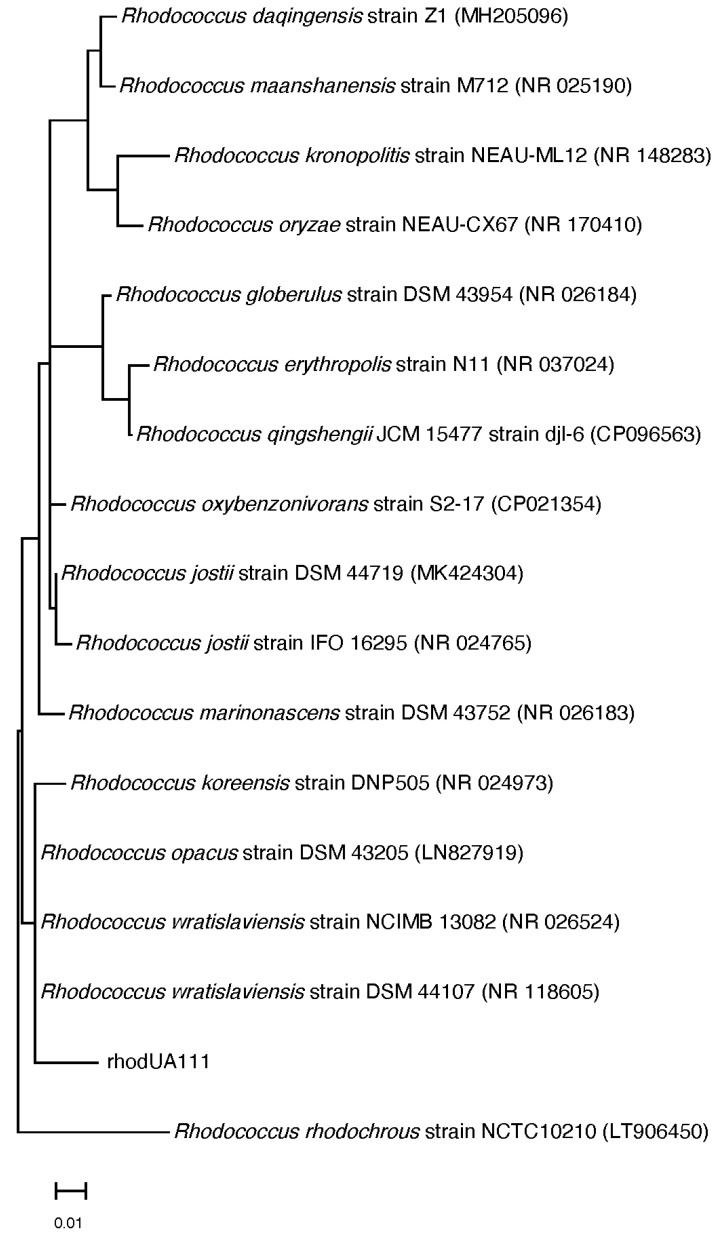
Phylogenetic tree of the 16S rRNA gene-marking *Rhodococcus* sp. UR111 isolate and its closest relatives.

The actinobacterium strains linked with Red Sea soft coral were isolated and taxonomically identified as *Rhodococcus* sp. UR111 based on morphology, 16S rRNA genome sequencing, and phylogenetic analysis (Figure 1).

### 3.2. Antimalarial Assay

As shown in Table 4, the activity of the culture extracts was tested in vitro against *Plasmodium falciparum.* The results revealed that the extract ISP2 exhibited the highest inhibitory activity, with IC_50_ values of 8.5 µg/mL, while Oligo was inactive when compared to the positive control drug chloroquine’s IC_50_ value (0.022 µg/mL).

### 3.3. Metabolomic Analysis

**Figure 2:**
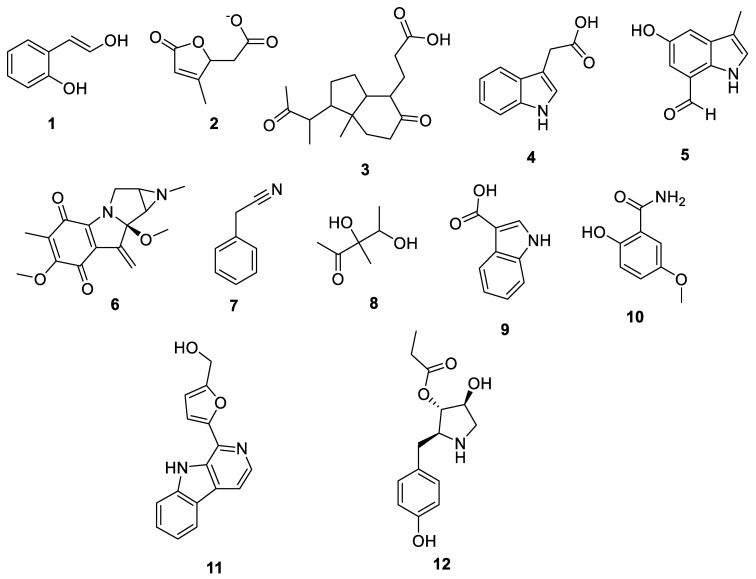
Metabolites dereplicated from *Rhodococcus* sp. crude extract.

Since marine actinomycetes are producing an increasing number of natural products, particularly species of *Streptomyces*, *Nocardia*, *Salinispora*, *Micromonospora*, and *Rhodococcus*, these microbes have also helped researchers find a number of clinically significant drugs. In addition, the obtained results of the antimalarial assay encouraged us to investigate the chemical profile of each of the tested extracts in order to identify the active antimalarial chemical components. Given this, metabolomic profiling of the actinomycete *Rhodococcus* sp. UA111 obtained from soft marine coral using LC-HR-MS for dereplication purposes has led to the identification of a number of metabolites, of which alkaloids were identified to predominate. LC-HRMS analysis was performed using both positive and negative ionization modes to detect the greatest number of metabolites possible. From the DNP database (Table 5 and Figure 2), the mass ion peak at *m/z* 135.0442 [M_H]_ (RT, 3.0118min) for the suggested molecular formulas C_8_H_8_O_2_ was identified as 2-(2′-Hydroxyphenyl) ethen-1-ol, which was previously obtained from *Rhodococcus benzothiophene* [12]. The mass ion peak at *m/z* 293.1755 [M_H]_ (RT, 4.668 min), in accordance with the molecular formula C_17_H_26_O_4_ was recognized as octahydro-7α-methyl-1-(1-methyl-2-oxo-propyl)-5-oxo-1H-indene-4-propanoic acid that was previously isolated from *Rhodococcus corallines* [13]. Whereas that at *m/z* 174.1022 [M_H]_ (RT, 2.6807min), corresponding to the molecular formula C_10_H_9_NO_2_, was suggested to be indole-3-acetic acid, which was previously isolated from *Rhodococcus* sp. BFI 332 [14]. The mass ion peak at *m/z* 155.0237 [M_H]_ (RT, 2.58059 min), in accordance with the molecular formula C_7_H_8_O_4_, was recognized as 2,5-Dihydro-3-methyl-5-oxo-2-furanacetic acid (S-form), which was previously isolated from *Rhodococcus rhodochrous* [10]. Additionally, another metabolite with the molecular formula C_10_H_9_NO_2_, for the mass ion peak at *m/z* 176.0712 [M_H]+ (RT, 4.1051 min), was characterized as rhodinodohyde, which was previously described from *Rhodococcus* sp. UA13 [3]. The metabolite, namely Mitomycin-K, with the molecular formula C_16_H_18_N_2_O_4_, was also dereplicated from the mass ion peak at *m/z* 303.134 [M_H]+ (RT, 2.684719 min); this compound was previously reported from *Rhodococcus* sp. UR59 [15]. Furthermore, the mass ion peak at *m/z* 116.0497 [M_H]_ (RT, 3.077 min) for the predicted molecular formula C_8_H_7_N was distinguished as 2-Phenylacetonitrile, which was isolated before from marine *Streptomyces* sp. GWS-BW-H5 [16]. The mass ion peak at *m/z* 131.0707 [M_H]_ (RT, 4.6038 min), for the suggested molecular formulas C_6_H_12_O_3_ was identified as 3,4-Dihydroxy-3-methylpentan-2-one that was previously obtained from *Streptomyces griseorubens* sp. ASMR4 [17]. Moreover, the mass ion peak at *m/z* 160.0395 [M_H]_ (RT, 3.08194 min), corresponding to the proposed molecular formula C_9_H_7_NO_2_, was identified as Indole-3-carboxylic acid, which was earlier obtained from *Streptomyces* sp. TK-VL_333 [18]. The mass ion peak at *m/z* 166.0503 [M_H]- (RT, 2.3045 min), with the molecular formula C_8_H_9_NO_3_, was recognized as 2-Hydroxy-5-methoxybenzamide that was previously isolated from marine *Streptomyces* sp. [19]. The mass ion peak at *m/z* 265.0973 [M_H]+ (RT, 3.67572 min), in accordance with the molecular formula C_16_H_12_N_2_O_2_, was recognized as Perlolyrin, which was previously isolated from a terrestrial *Streptomyces* sp. GW11/1695 [20] and a marine *Streptomyces* sp. [21] The metabolite, 3097-B2, with the molecular formula C_14_H_19_NO_4_, was also dereplicated from the mass ion peak at *m/z* 264.1238 [M_H]_ (RT, 2.9919 min); this compound was previously reported from marine *Streptomyces* sp. [22].

### 3.4. Multivariate Data Analysis

After being processed by the mzmine software, an MVA on the gathered LC-HRMS data was carried out in order to condense the enormous amount of data obtained and identify correlation and distinction among the tested samples. The LC-MS analysis dataset was statistically treated using MetaboAnalyst 5.0 [23,24].

#### 3.4.1. Unsupervised Analysis

**Figure 3:**
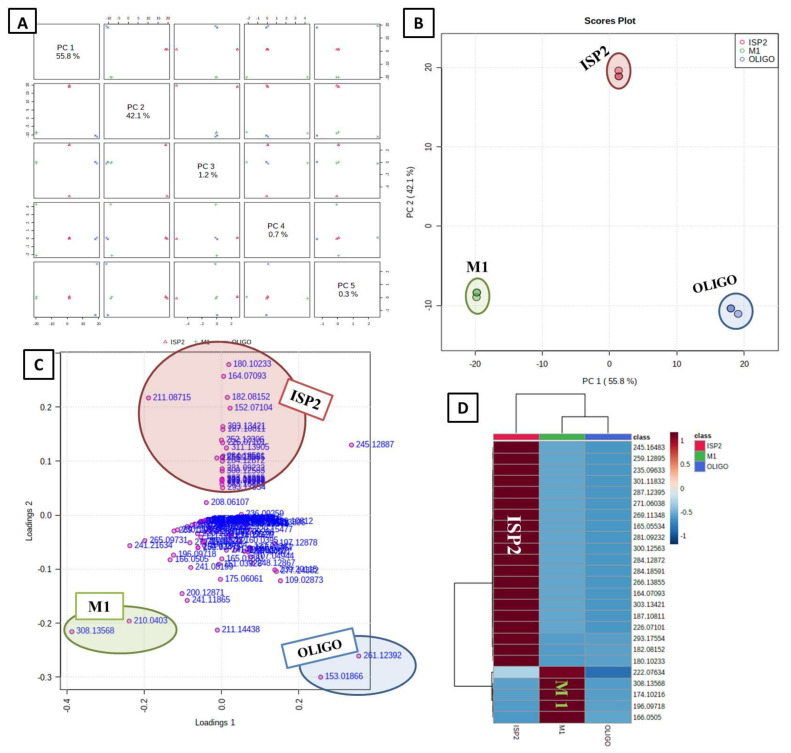
Metabolomics multivariate analysis. (**A**) PCA pairwise score plot of the unsupervised method; (**B**) 2D PCA scores plot of the unsupervised method; (**C**) 2D PCA loadings plot of the unsupervised method; (**D**) Heatmap plot.

As an initial “blind” step, the unsupervised principal component analysis (PCA) method was implemented first. As shown in the PCA pairwise score plots (Figure 3A), there are five PCA components (PCs) that explained 100.1% of the total variation, in which the first and second PCs separately contributed to 97.9% of the total variation (PC1 and PC2 represent 55.8% and 42.1%, respectively) (Figure 3A). The samples were primarily allocated to three distinct areas between PC1 and PC2, which revealed statistically significant differences between the three extracts, indicating their chemical variation in the PCA 2D scores plot (Figure 3B). On the other hand, the heat map plot (Figure 3D) showed distinct patterns for the culture extracts ISP2 and M1. The metabolites (*m/z*) that contributed to the fluctuation of the anomalous samples were shown by the PCA loadings plot (Figure 3C); the unique clusters those metabolites (*m/z*) formed in the loading plot correspond to the locations of the anomalous samples in the scores plot. Such metabolites were dereplicated using the Dictionary of Natural Products (DNP); the annotated discriminatory compounds for ISP2 corresponding to *m/z* (retention time in min) 180.10233 [M_H]^+^ (2.4039) was identified as p-tolyl-3-aminopropanoate (C_10_H_13_NO_2_) [25]. The mass ion peak at *m/z* 164.07093 [M_H] (RT, 1.95985 min), corresponding to the proposed molecular formula C_9_H_11_NO_2_, was identified as Anthranilic acid ethyl ester [26]. The mass ion peak at *m/z* 182.08152 [M_H]^+^ (RT, 2.997325 min), in accordance with the molecular formula C_9_H_11_NO_3_, was recognized as tyrosol carbamate [27]. In contrast, that at *m/z* 152.07104 [M_H]^−^ (RT, 2.9552 min) corresponding to the molecular formula C_8_H_9_NO_2_, was suggested to be streptokordin [28]. The mass ion peak at *m/z* 211.08715 [M_H]^+^ (RT, 3.872317 min), in accordance with the molecular formula C_13_H_10_N_2_O, was identified as penicinoline E [29]. On the other hand, the annotated discriminatory compounds for M1, corresponding to *m/z* (retention time in min) 308.13568 [M_H]^+^ (2.36095) and 210.0403 [M+H]^−^ (2.304102) were putatively identified as antibiotic A 53868A (C_11_H_22_N_3_O_5_P) and antibiotic T0007 B_2_ (C_9_H_9_NO_5_) [30], respectively.

#### 3.4.2. Supervised Analysis

**Figure 4:**
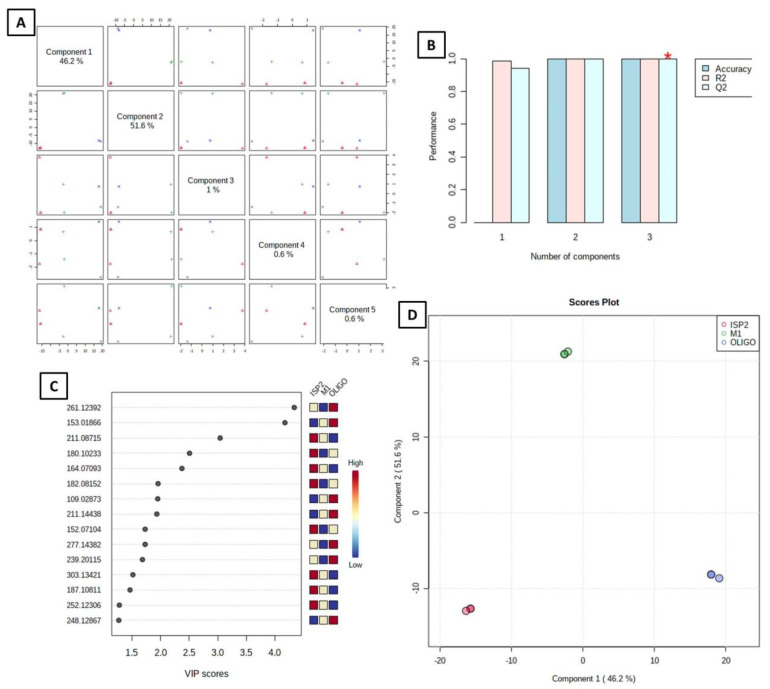
Metabolomics multivariate analysis. (**A**) PLS pairwise score plot; (**B**) 2D PCA scores plot of the unsupervised method; (**C**) 2D PCA loadings plot of the unsupervised method; (**D**) Heatmap plot.

The supervised approach frequently employed in metabolomics studies for classification and supervised biomarker discovery, partial least squares discriminant analysis (PLS-DA), makes use of multivariate linear regression techniques [31]. Both the prediction and the performance of the developed model were good (predictive power of models, Q2 = 1.0; model goodness, R2 = 1.0); a high Q2 value indicates good predictions (Q2 values were those that were very near to 1.0) (Figure 4B). The differences between the samples were confirmed after PLS-DA supervised analysis; in the PLS-DA 2D paired score plot (Figure 4A), five components accounted for 100 percent of the total variation. Two PLS components (PCs) explained 97.8% of the overall variances in the PLS-DA 2D scores plot, with the first and second PCs contributing 46.2 and 51.6 percent, respectively (Figure 4D). One of the crucial measurements in PLS-DA is the variable Importance in Projection (VIP) (Figure 4C), which is shown in Figure 4C. The top 15 most significant traits with the highest value, according to PLS-DA, were demonstrated by VIP. The top 15 significant metabolites (*m/z*) were listed on the vertical axis in descending order according to the scores they received on the horizontal axis, and the relative concentrations of the important metabolites in the tested samples are shown in the attached colored box on the right (Figure 4B) [32].

The observed variations emphasize how crucial it is to use a diverse range of media to improve the isolation effectiveness of metabolites from actinomycetes associated with the marine environment. Based on prior experience and published reports, the actinomycete cultivation media M1, ISP2, and OLIGO were selected [2]. The results showed that ISP2 media was preferred for growing the genus *Rhodococcus* and the production of bioactive antimalarial compounds. 

### 3.5. Molecular Docking Simulations

**Figure 5:**
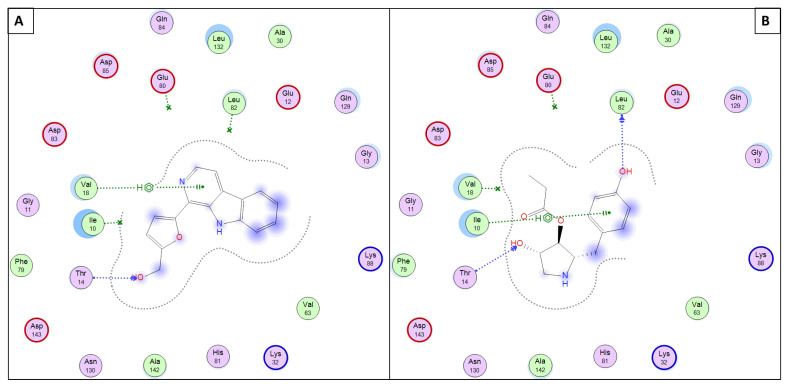
Presumptive mode of interaction of molecules 11 and 12 within active site of *P. falciparum* kinase (PDB ID: 1V0P); (**A**) Perlolyrine (11); (**B**) 3097-B2 (12).

**Figure 6:**
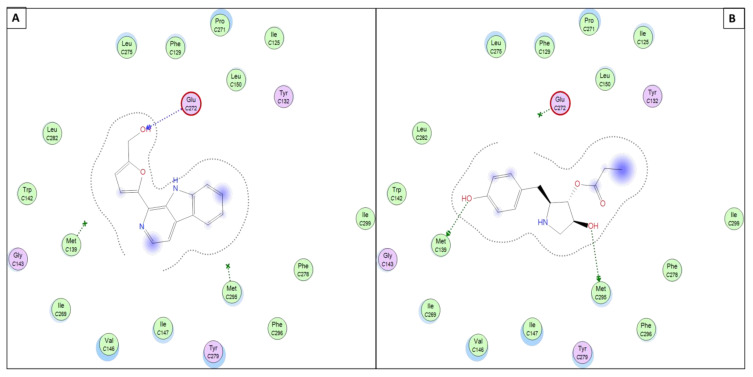
Presumptive mode of interaction of molecule 11 (**A**) and 12 (**B**) within active site of *P. falciparum* mitochondrial cytochrome bc1 complex (PDB ID: 4PD4). showing H-bond in blue and/or green arrows and solvent accessible contour as black-dotted line around whole molecule.

**Figure 7:**
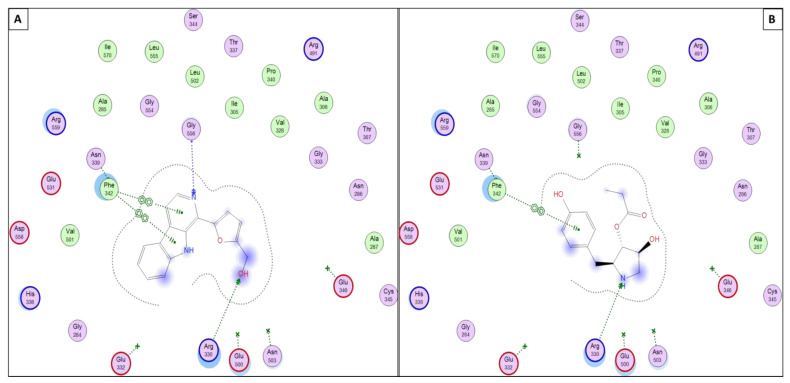
Presumptive mode of interaction of molecules 11, 12, and 9X0 within active site of *P. falciparum* lysyl-tRNA synthetase (PfKRS1; PDB ID: 6AGT). (**A**) Perlolyrine (11); (**B**) 3097-B2 (12).

From our interest to explore the possible cellular targets upon which these dereplicated molecules could affect within *P. falciparum* species, we ran molecular docking simulations within the crystal structures of various proteins found in *P. falciparum* species, such as the *Plasmodium falciparum* cell cycle regulator and non-human-derived cyclin-dependent kinase, called *P. falciparum* kinase (PfPK5; PDB ID: 1V0P). Additionally, the *P. falciparum* cytochrome bc1 complex (Cyt bc1; PDB ID: 4PD4), and finally, *P. falciparum* lysyl-tRNA synthetase (PfKRS1; PDB ID: 6AGT). These simulations revealed a moderate to strong docking score (S = −4.45 to −7.11 kcal/mol) with all test molecules within the three active sites used in this study (as listed in Table 1). Molecules 11 (Perlolyrine) and 12 (3097-B2) showed the best docking profile among all test molecules as found with their docking score and binding interactions with various amino acid residues lining the proteins’ active sites (as illustrated in Figure 5, Figure 6 and Figure 7 and Figures S1–S3 in the Supporting Information File).

Briefly, in silico molecular docking simulations within the active site of *P. falciparum’s* kinase (PDB ID: 1V0P) revealed that both molecules 11 and 12 succeed in having binding interactions (either H-bonding or pi-H interactions, as listed in Table 2) with at least one of the key amino acid residues (VAL18, THR14, LEU82, ILE10, and/or LYS88) lining this active site, as shown in Figure 5.

Moreover, both molecules 11 and 12 showed an interesting binding mode within the *P. falciparum* mitochondrial cytochrome bc1 complex (PDB ID: 4PD4) active site mediated through H-bond donor and acceptor interactions with key amino acid residues lining the active site (GLU272, MET295, and/or MET139), as listed in Table 2. Such types of interactions provided better stabilization of the molecules within the target protein and could be used as a scaffold for the further development of antimalarial agents (as shown in Figure 6).

Finally, the docking scores and the docking accuracy of the test molecules within the crystal structure of *P. falciparum* lysyl-tRNA synthetase (PfKRS1; PDB ID: 6AGT) showed the highest and best possibility of being a target by such compounds and could be used to illustrate their antimalarial biological activities. In brief, the docking simulations within lysyl-tRNA synthetase resulted in the best docking score and number of binding interactions within the active site of lysyl-tRNA synthetase crystal structures, especially with molecules 11 and 12, which have a high and comparable docking score within all other dereplicated molecules and to the co-crystallized ligand (9X0), as shown in Table 2 and Figure 7.

#### 3.5.1. ADME Parameters and Drug-likeness Computational Analysis

Investigating the oral bioavailability of such dereplicated molecules was achieved by measuring the physicochemical descriptors, pharmacokinetics, drug-like nature, and medicinal chemistry friendliness of six of these dereplicated molecules (3, 4, 5, 6, 11, and 12) using the SwissADME free web tool (http://www.swissadme.ch/ accessed on 7 August 2022). Starting with the role of five (Lipinski’s RO5) [33,34], all of the test molecules have values for physicochemical within the required ranges of candidate drugs, as shown in Table 3.

Briefly, having H-bonding centers (either acceptor or donor) helps in enhancing water solubility and H-bond formation with various amino acid residues lining the target’s active sites, in the same way, having an acceptable number of rotating bonds helps in the adaptation and flexibility alignment of such molecules within the target active sites. Additionally, having a partition coefficient (i.e., lipophilicity parameter) within −0.5 to ≤5, and both TPSA and MR values below 140 and 130, respectively, generally indicates a good probability of penetration through cell membranes, gastrointestinal penetration, and hence bioavailability. Fortunately, all of the test molecules (except molecule 11) were found to not be a substantial substrate for permeability glycoprotein (also known as multidrug resistance protein, which is responsible for the efflux of drugs out of cells and hence reduces their pharmacological efficacy) and having an Abbott oral bioavailability score above zero, indicating their high probability of medicinal impact and biological activities in clinical trials [35]. 

Finally, all the test molecules pass the medicinal chemistry filters of Lipinski. In the same way, all the test molecules also pass (except molecule 6) the Pan-assay of interference compounds (PAINS), indicating their high probability of being a drug candidate for drug discovery and development, as shown in Table 3.

#### 3.5.2. Perlolyrine (11) and 3097-B2 (12)

Perlolyrine (11) is a β-carboline alkaloid that was previously isolated from the roots of *Codonopsis pilosula.* It was also reported in rye grass (*Lolium perenne* L.) [36], *Codonopsis lanceolata*, *Panax gingseng* [37], and recently isolated from a terrestrial *Streptomyces* species [20]. On the other hand, 3097-B2 (12) is a pyrrolidine derivative structurally relative to the antifungal drug anisomycin; it was previously isolated from the culture broth of *Streptomyces* strain SA3097 and showed cytotoxic activity in vitro against the cancer cell lines, human lung carcinoma (LU99) and human breast cancer (MCF-7) [22]. Moreover, Bhattarai et al. identified 3097-B2 (12) in the fermented culture of *Streptomyces* species isolated from the soils of Nepal using an untargeted metabolomics study [38].

## 4. Conclusions

*Rhodococcus* sp., an infrequently studied marine bacterium, was investigated utilizing the established process using LC-HR-ESI-MS-based analytical techniques. Herein, the OSMAC technique was used to isolate and subculture the actinomycete, *Rhodococcus* associated with the Red Sea soft coral *Nephthea* sp. in three different media (M1, ISP2, and OLIGO). The extracts were then examined for their antimalarial activity and chemical diversity. The ISP2 extract showed substantial antimalarial efficacy. On the other hand, several metabolites of various structural kinds have been identified as a result of our study of this species, belonging to different classes, mainly alkaloids. In silico studies revealed that the metabolites perlolyrine (11) and 3097-B2 (12) have a high and comparable docking score within all other dereplicated molecules. This study emphasized the unique function of actinomycetes associated with marine organisms as an unexplored source of active metabolites for the creation of effective natural antimalarial drugs. By combining the analytical, statistical, and dereplication approaches for examining their bioactive principles, it is possible to effectively shorten or replace the time-consuming bioassay-guided isolation schemes and complex chromatographic procedures. Biochemometrics approaches have been widely used in natural product research as an initial evaluation step in the process of finding new drugs from actinomycetes. Targeting the bioactive antimalarial metabolites in complicated crude extracts can therefore be more successful with greater integration with in silico technologies such as neural network-based virtual screening. Using elicitation approaches, cryptic gene clusters in microorganisms could be a new approach to finding new antimalarial drug leads.

## Figures and Tables

**Table 1:** Docking simulations of dereplicated molecules and co-crystallized ligands within three of *P. falciparum* proteins.

#	Cpd ID	Isolated Molecules	1V0P ^a^		4PD4 ^b^		6AGT ^c^	
			S ^g^	RMSD ^h^	S	RMSD	S	RMSD
1	3	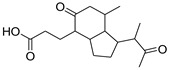 Indene propanoic acid derivative	−5.74	1.67	−5.55	1.97	−5.88	0.92
2	4	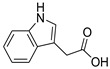 Indole-3-acetic acid	−4.45	1.40	−5.16	1.77	−5.15	1.10
3	5	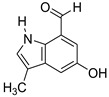 Rhodinodohyde	−5.01	0.96	−4.85	0.81	−5.37	1.53
4	6	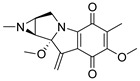 Mitomycin-K	−5.39	1.68	−5.37	0.73	−6.09	1.16
5	11	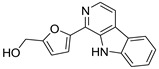 Perlolyrine	−5.98	1.04	−5.84	1.69	−6.91	1.12
6	12	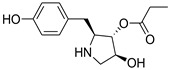 3097-B2	−5.89	1.99	−6.50	1.56	−7.11	1.97
Co-crystallized Ligand			−7.32 ^d^	1.21	−6.45 ^e^	1.64	−7.98 ^f^	0.63

^a^ 1V0P: *P. falciparum* kinase; ^b^ 4PD4: cytochrome bc1; and ^c^ 6AGT: lysyl-tRNA synthetase (PfKRS1) active sites; ^d^ Purvalanol B (PVB); ^e^ 2-[trans-4-(4-chlorophenyl)cyclohexyl]-3-hydroxynaphthalene-1,4-dione (AOQ); ^f^ N-(cyclohexylmethyl)-4-oxo-4H-1-benzopyran-2-carboxamide (9X0); ^g^ Docking Score (S; Kcal/mol); ^h^ Root-Mean-Square Deviation (Å).

**Table 2:** Binding interactions of dereplicated molecules 11 and 12 within three *P. falciparum* proteins.

Molecule ID	1V0P ^a^			4PD4 ^b^			6AGT ^c^		
	Type	a.a. Residue	Distance ^e^	Type	a.a. Residue	Distance	Type	a.a. Residue	Distance
11	H-acceptor	THR14	3.20	H-acceptor	GLU272	3.49	H-acceptor	GLY556	3.46
							H-acceptor	ARG330	3.15
	Pi-H	VAL18	4.21				Pi-Pi	PHE342	3.81
							Pi-Pi	PHE342	3.83
12	H-donor	LEU82	2.90	H-donor	MET295	3.33	H-acceptor	ARG330	3.15
	H-acceptor	THR14	2.86	H-donor	MET139	3.83	Pi-Pi	PHE342	3.90
	Pi-H	ILE10	3.90						
Co-crystallized Ligands ^d^	H-donor	GLU80	3.17	Pi-H	ILE 125	4.15	H-acceptor	ASN339	3.05
	H-donor	LEU82	2.76						
	H-acceptor	LEU82	3.01				Pi-Pi	PHE342	3.58
	Ionic	LYS88	3.91						
	Pi-H	VAL18	4.01						

^a^ 1V0P: *P. falciparum* kinase; ^b^ 4PD4: cytochrome bc1 complex; and ^c^ 6AGT: lysyl-tRNA synthetase (PfKRS1) active sites; ^d^ Co-crystallized ligands: PVB for 1V0P, AOQ for 4PD4, and 9X0 for 6AGT; ^e^ Bond distance (Å).

**Table 3:** ADMET, Pharmacokinetic and PAINS analysis of dereplicated molecules (3, 4, 5, 6, 11, and 12).

ID	M.F. ^a^	M.W. ^b^	Nrotb ^c^	HBA ^d^	HBD ^e^	MR ^f^	TPSA ^g^	iLogP ^h^	Water Solubility	HIA% ^i^	BBB Permeant ^j^	Pgp Substrate ^k^	F ^l^	PAINS ^m^
3	C17H26O4	294.39	5	4	1	81.78	71.44	2.16	Soluble	High	Yes	No	0.85	0
4	C10H9NO2	175.18	2	2	2	49.84	53.09	1.01	Soluble	High	Yes	No	0.85	0
5	C10H9NO2	175.18	1	2	2	50.68	53.09	1.08	Soluble	High	Yes	No	0.55	0
6	C16H18N2O4	302.33	2	5	0	85.38	58.85	2.82	Very soluble	High	No	No	0.55	1
11	C16H12N2O2	264.28	2	3	2	77.43	62.05	2.36	Soluble	High	Yes	Yes	0.55	0
12	C14H19NO4	265.3	5	5	3	74.13	78.79	2.11	Soluble	High	No	No	0.55	0
RO5		≤500	≤10	≤10	≤5	≤130	≤140	≤5						

^a^ MF, Molecular Formula; ^b^ M.W., Molecular Wight; ^c^ nrotb, # of rotatable bonds; ^d^ HBA, Hydrogen Bond Acceptor; ^e^ HBD, Hydrogen bond donor; ^f^ MR, Molar Refractivity; ^g^ TPSA, Total Polar Surface Area; ^h^ iLogP, octanol/water partition coefficient; ^i^ HIA%, human gastrointestinal absorption; ^j^ BBB permeant, Blood–brain barrier penetration; ^k^ Pgp substrate, Permeability glycoprotein; ^l^ F, Abbott oral bioavailability score; ^m^ PAINS, Pan-Assay Interference Compounds.

**Table 4:** In Vitro antimalarial activity of *Rhodococcus* fermented extracts against *Plasmodium falciparum*.

Sample	IC50 (µg/mL)
M1	24.7
ISP2	8.5
Oligo	>50
Chloroquine	0.022

**Table 5:** Metabolites dereplicated from *Rhodococcus* crude extract.

#	Ionization	*m/z*	Rt	Name	Formula	Key Fragments	Source	Reference
1	N	135.0442	3.0118	2-(2′-Hydroxyphenyl) ethen-1-ol	C_8_H_8_O_2_		*Rhodococcus benzothiophene*	[12]
2	N	155.0237	2.580592	2,5-Dihydro-3-methyl-5-oxo-2-furanacetic acid (S-form)	C_7_H_8_O_4_		*Rhodococcus rhodochrous*	[10]
3	N	293.1755	4.668813	Octahydro-7a-methyl-1-(1-methyl-2-oxo-propyl)-5-oxo-1H-indene-4-propanoic acid	C_17_H_26_O_4_	221.13	*Rhodococcus corallinus*	[13]
4	N	174.1022	2.6807	Indole-3-acetic acid	C_10_H_9_NO_2_		*Rhodococcus sp. BFI 332*	[14]
5	P	176.0712	4.105067	Rhodinodohyde	C_10_H_9_NO_2_	72.07	*Rhodococcus sp. UA13*	[3]
6	P	303.1342	2.684719	Mitomycin-K	C_16_H_18_N_2_O_4_	257.06	*Rhodococcus sp. UR59*	[15]
7	N	116.0497	3.077592 2.708363	2-Phenylacetonitrile	C_8_H_7_N		*marine Streptomyces sp. GWS-BW-H5*	[16]
8	N	131.0707	4.603822	3,4-Dihydroxy-3-methylpentan-2-one	C_6_H_12_O_3_	87.04	*Streptomyces griseorubens sp. ASMR4*	[17]
9	N	160.0395	3.081942	Indole-3-carboxylic acid	C_9_H_7_NO_2_		*Streptomyces sp. TK-VL_333*	[18]
10	N	166.0503	2.304583	2-Hydroxy-5-methoxybenzamide	C_8_H_9_NO_3_		*marine Streptomyces*	[19]
11	P	265.0973	3.675722	Perlolyrin	C_16_H_12_N_2_O_2_	264.87, 165.64	*Streptomyces sp. isolate GW11/1695*	[20]
							*Marine streptomyces*	[21]
12	N	264.1238	2.991953	3097-B	C_14_H_19_NO_4_		*marine Streptomyces*	[22]

## Data Availability

The data are contained within the article or Supplementary Material.

## Supplementary Materials

The following are available online at https://www.mdpi.com/article/10.3390/antibiotics11111631/s1, Figure S1. Presumptive mode of interaction of molecules 3, 4, 5, 6, and PVB within the active site of *P. falciparum* kinase (PDB ID: 1V0P). Figure S2. Presumptive mode of interaction of molecules 3, 4, 5, 6, and AOQ within the active site of *P. falciparum* mitochondrial cytochrome bc1 complex (PDB ID: 4PD4). Figure S3. Presumptive mode of interaction of molecules 3, 4, 5, 6, and 9X0 within the active site of *P. falciparum* lysyl-tRNA synthetase (PfKRS1; PDB ID: 6AGT).
