# Supplementary material for: Metabolomic Profiling, In Vitro Antimalarial Investigation and In Silico Modeling of the Marine Actinobacterium Strain Rhodococcus sp. UR111 Associated with the Soft Coral Nephthea sp
Source: Antibiotics (Basel). 2022 Nov 15;11(11):1631. doi: 10.3390/antibiotics11111631 (PMC9686727; doi:10.3390/antibiotics11111631)
Supplement: Supplementary file 1 [file antibiotics-11-01631-s001.zip › antibiotics-1976616-supplementary.pdf]

Supporting Information

Molecular Docking Simulations

Indene propanoic acid **derivative (Molecule 3)**

Indole-3-acetic acid **(Molecule 4)**

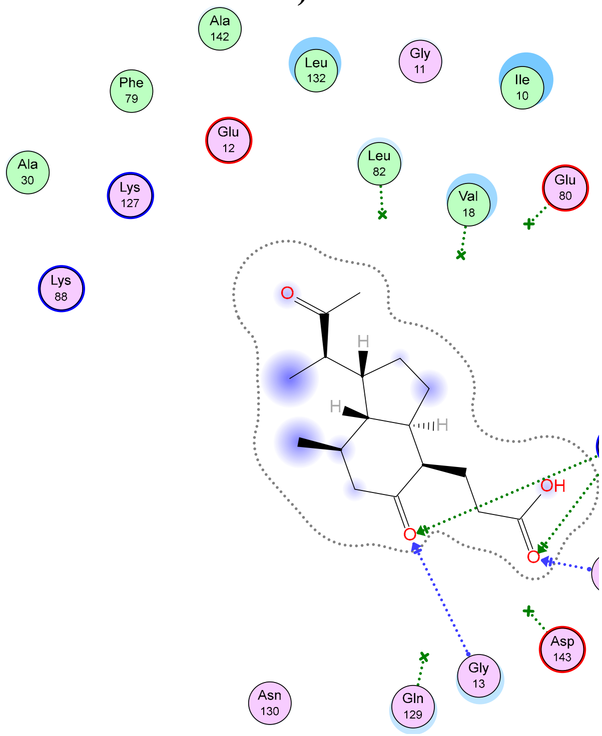

Rhodinodohyde **(Molecule 5)**

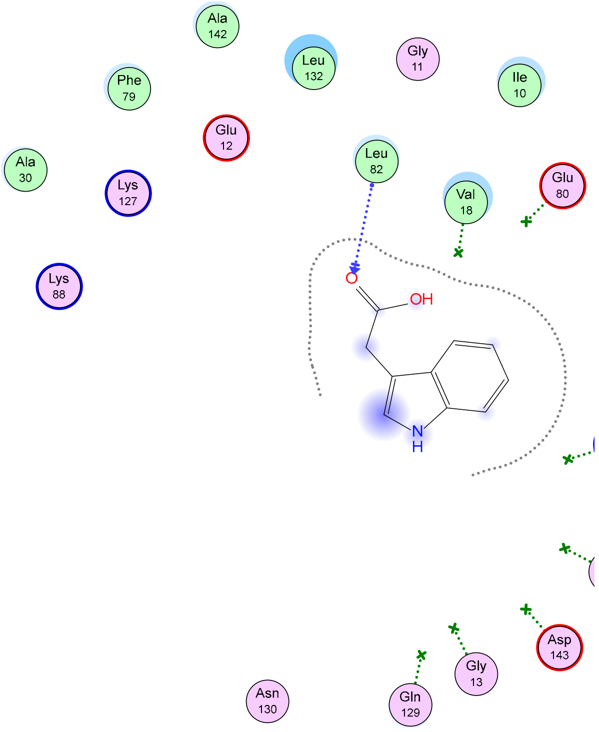

Mitomycin-K **(Molecule 6)**

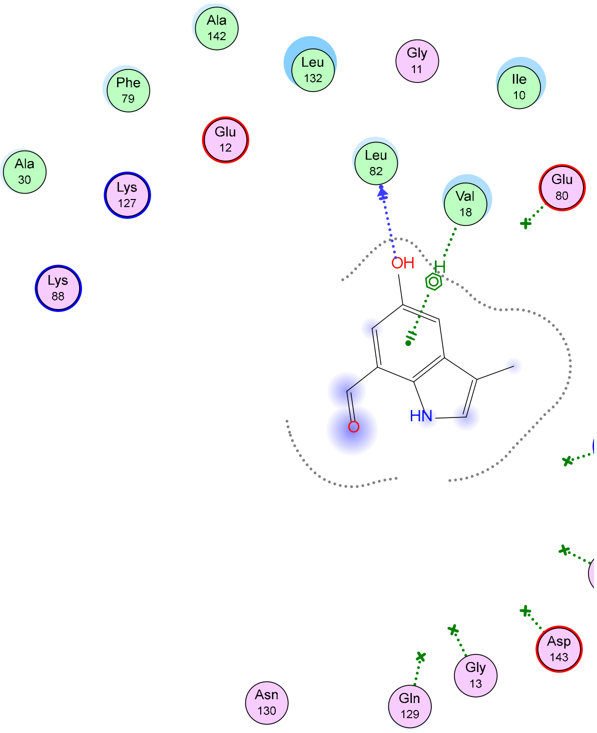

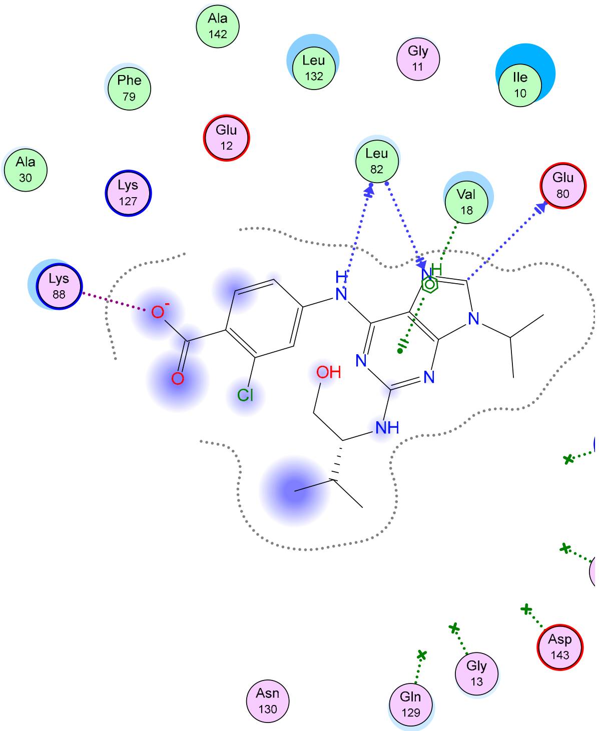

PVB (Co-crystallized ligand)

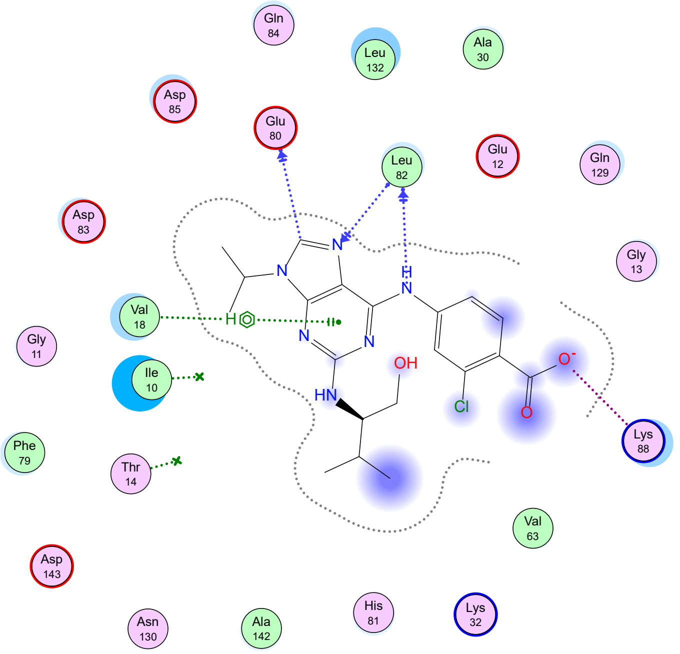

Figure S1. Presumptive mode of interaction of molecules **3**, **4**, **5**, **6**, and **PVB** within active site of *P. falciparum* kinase (PDB ID: 1V0P)

Indene propanoic acid derivative **(Molecule 3)**

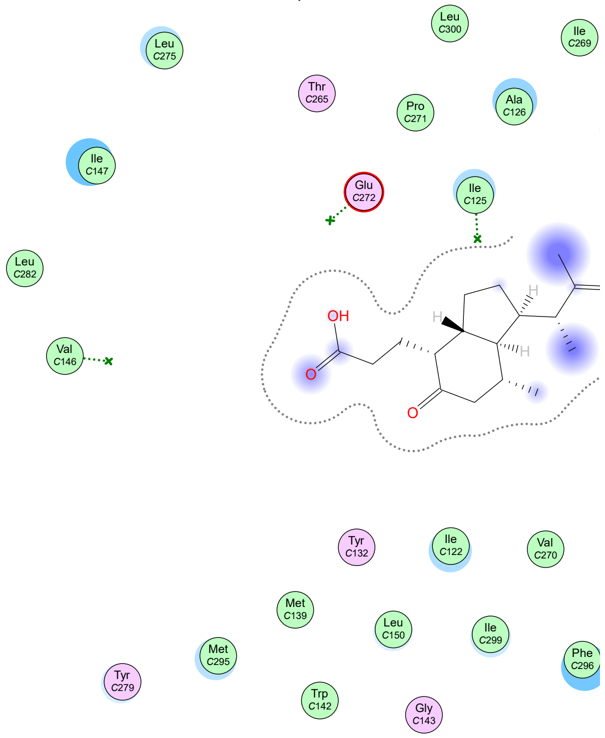

Indole-3-acetic acid **(Molecule 4)**

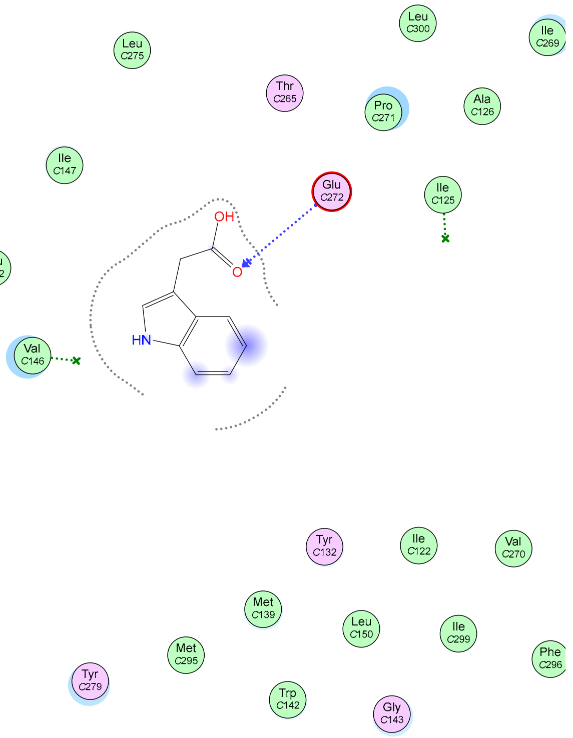

Rhodinodohyde **(Molecule 5)**

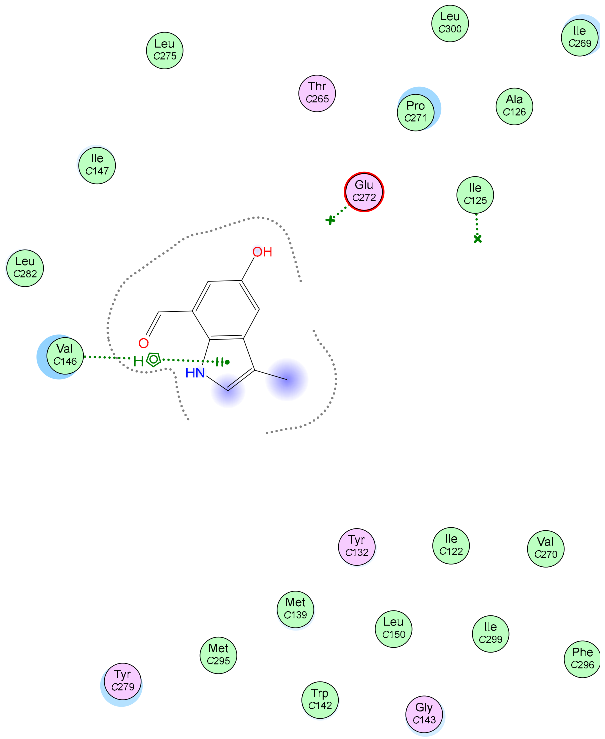

Mitomycin-K **(Molecule 6)**

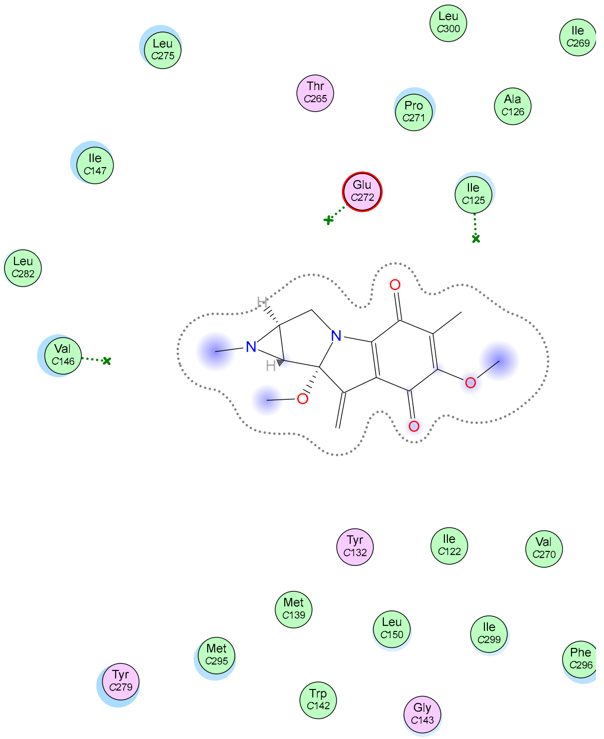

AOQ

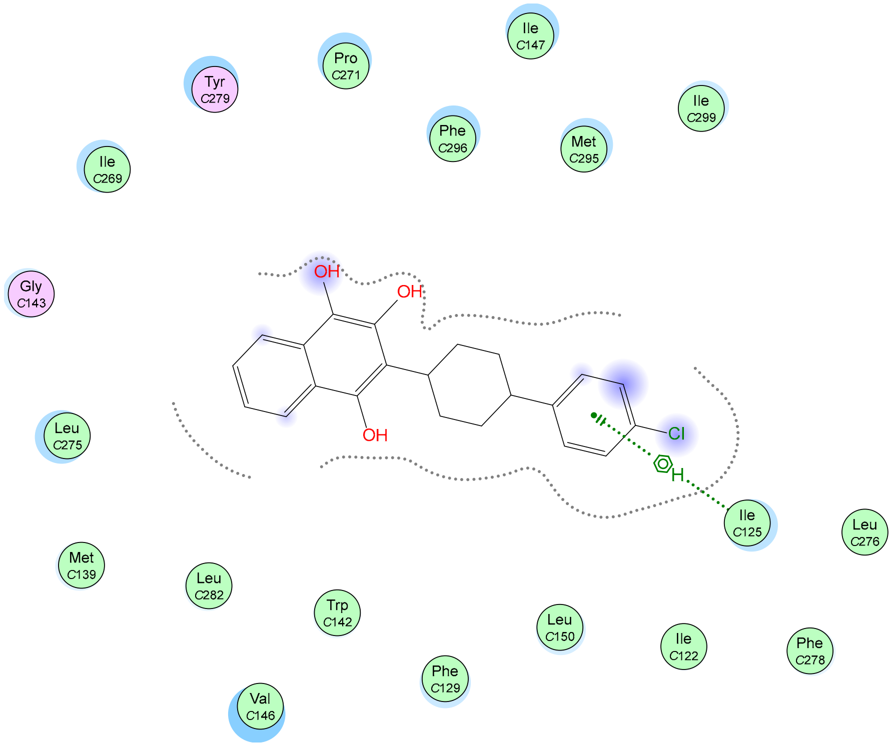

Figure S2. Presumptive mode of interaction of molecules **3**, **4**, 5, 6, and **AOQ** within active site of *P. falciparum* mitochondrial cytochrome bc1 complex (PDB ID: 4PD4).

Indene propanoic acid derivative **(Molecule 3)**

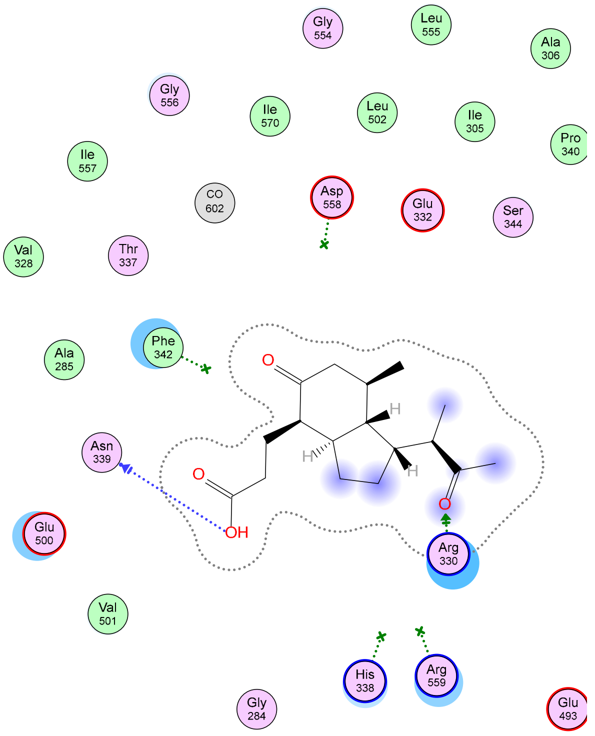

Indole-3-acetic acid **(Molecule 4)**

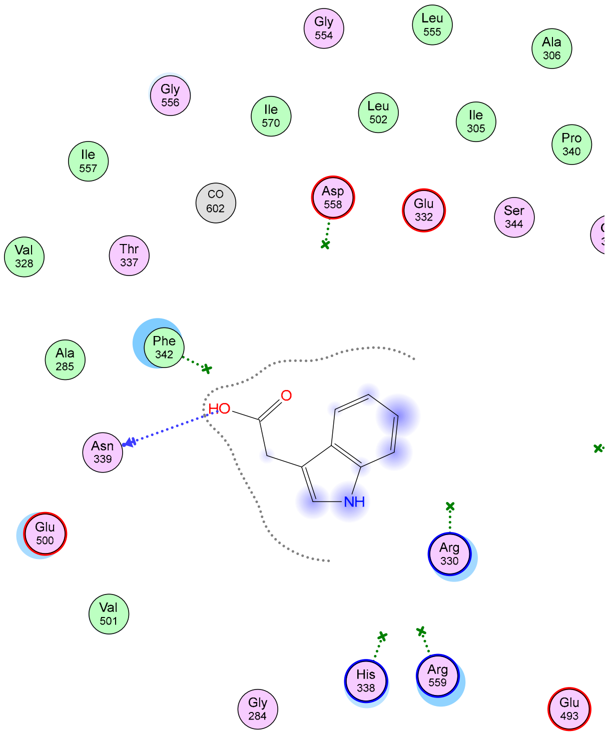

Rhodinodohyde **(Molecule 5)**

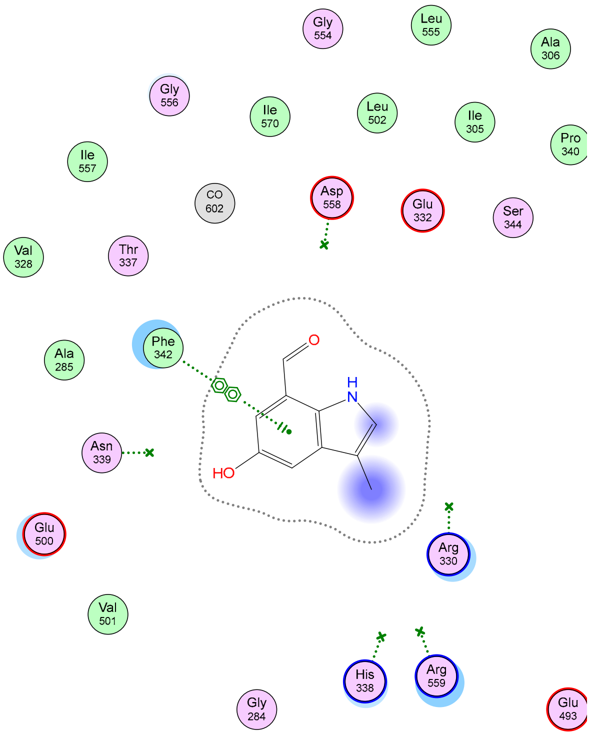

Mitomycin-K **(Molecule 6)**

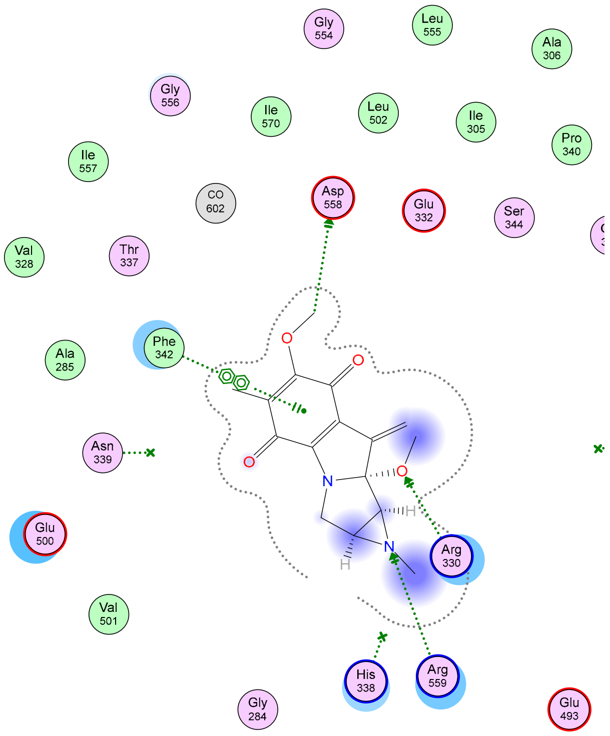

9X0

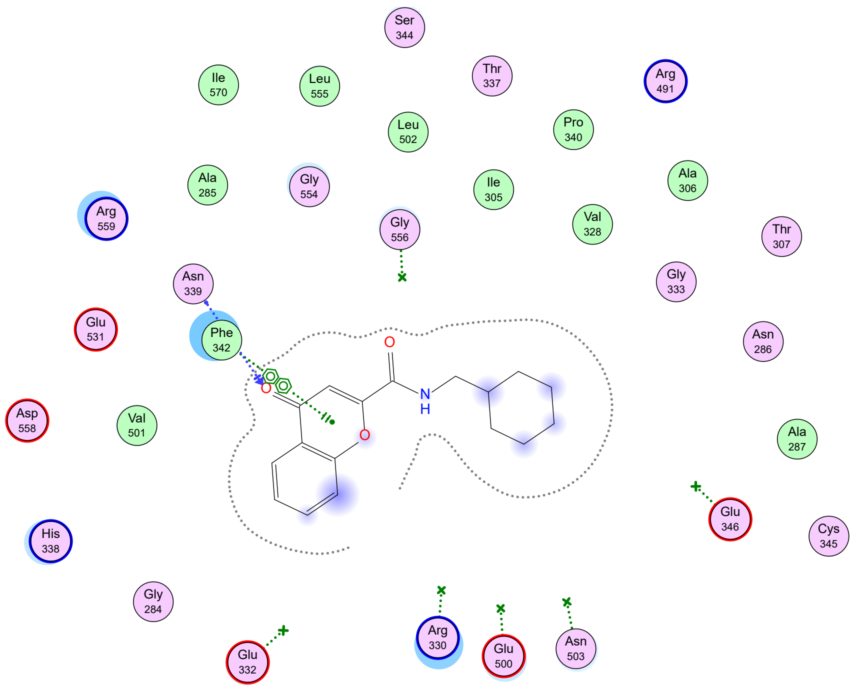

Figure S3. Presumptive mode of interaction of molecules **3**, **4**, **5**, **6**, and **9X0** within active site of *P. falciparum* lysyl-tRNA synthetase (PfKRS1; PDB ID: 6AGT)
